# Tryptophan-enriched diet or 5-hydroxytryptophan supplementation given in a randomized controlled trial impacts social cognition on a neural and behavioral level

**DOI:** 10.1038/s41598-021-01164-y

**Published:** 2021-11-04

**Authors:** V. Zamoscik, S. N. L. Schmidt, R. Bravo, L. Ugartemendia, T. Plieger, A. B. Rodríguez, M. Reuter, P. Kirsch

**Affiliations:** 1 Department of Clinical Psychology, Central Institute of Mental Health, Medical Faculty Mannheim, Heidelberg University, J 5, 68159 Mannheim, Germany; 2 Department of Psychology, Laboratory of Neurogenetics, University of Bonn, Bonn, Germany; 3 Research Group of Clinical Psychology, Department of Psychology, University of Konstanz, Konstanz, Germany; 4 Chrononutrition Laboratory, Neuroimmunephysiology and Chrononutrition Research Group, Faculty of Science, University of Extremadura, Badajoz, Spain

**Keywords:** Neuroscience, Psychology

## Abstract

Understanding of emotions and intentions are key processes in social cognition at which serotonin is an important neuromodulator. Its precursor is the essential amino acid tryptophan (TRP). Reduced TRP availability leads to weaker impulse control ability and higher aggression, while TRP supplementation promotes confidence. In a double-blind placebo-controlled fMRI study with 77 healthy adults, we investigated the influence of a 4 week TRP enriched diet and an acute 5-hydroxytryptophan (5-HTP) intake on two social-cognitive tasks, a moral evaluation and an emotion recognition task. With 5-HTP, immoral behavior without negative consequences was rated as more reprehensible. Additionally, during story reading, activation in insula and supramarginal gyrus was increased after TRP intake. No significant effects of TRP on emotion recognition were identified for the whole sample. Importantly, emotion recognition ability decreased with age which was for positive emotions compensated by TRP. Since the supramarginal gyrus is associated with empathy, pain and related information integration results could be interpreted as reflecting stricter evaluation of negative behavior due to better integration of information. Improved recognition of positive emotions with TRP in older participants supports the use of a TRP-rich diet to compensate for age related decline in social-cognitive processes.

## Introduction

Emotion recognition and understanding of emotions and intentions are key factors in social cognition^1^. Especially in morally challenging situations, the ability of integrating relevant information, dealing with own affective reactions, perspective-taking, and feeling empathy is crucial^2,3^.

A major neuromodulator in regulating affective and social-cognitive processes is serotonin^4^ the precursor of which is the essential amino acid tryptophan (TRP). TRP is the scarce amino acid in many foods, however, abundant in e.g. soy, cashew, cacao, and whey products (Food Composition Database SR-26 of the United States Department of Agriculture: https://ndb.nal.usda.gov/ndb/search/list). TRP and other large neutral amino acids (LNAA) like tyrosine compete at the blood brain barrier^5^ which leads to lower brain TRP levels when more other LNAAs are consumed. As serotonin itself cannot cross the blood brain barrier, it is necessary that a sufficient amount of TRP or 5-hydroxytryptophan (5-HTP) is available in the brain. 5-HTP is the direct precursor of serotonin and is almost completely metabolized to serotonin showing a plasma half-life of about 4–6 h^6,7^. 5-HTP can be found in food, e.g. the bean *Griffonia simplicifolia*^8^, or can be synthesized from TRP by the enzyme tryptophan hydroxylase, which is the initial and rate-limiting step in the synthesis of serotonin. TRP is metabolized to about 95% via the kynurenine pathway and to about 5% via the serotonin pathway^9^ mainly in the gut. Serotonin can be converted into melatonin during darkness or it is excreted in the urine in the form of 5-hydroxyindoleacetic acid (5-HIAA). A daily intake of about 4–6 mg TRP/kg is recommended for adults^10^. Supplementation of TRP increases plasma TRP^11^ and improves the release of serotonin in the rat brain^12^.

So far, the effect of TRP on social cognition has been mainly investigated in the context of TRP depletion revealing heterogeneous results. While some studies found TRP depletion to impair the recognition of fear in healthy individuals^13^ and the recognition of happiness in remitted depression^14^, others found no effects in participants with a family history of depression^15^. Further, TRP depletion leads to lower impulse control ability and higher aggression levels^16^ whereas TRP supplementation seems to reduce aggression and to promote prosocial behavior including interpersonal trust and charitable donating^17–19^. It has been argued that the effect of TRP depletion and supplementation acts via serotonin availability on a dimension from agreeability to quarrelsomeness and via that influence mood and social interactive behavior^20^. An important facet of prosocial behavior which is negatively related to aggression and quarrelsomeness is empathy^21^.

Empathy supports our ability to understand the intentions, beliefs, and needs of others. Usually, research discriminates affective and cognitive empathy^22^. While empathizing with the sensations of another person is called affective empathy, cognitive empathy is characterized by perspective-taking^23,24^. Cognitive as well as emotional aspects of empathy have been associated to moral decision-making^25^ supporting the idea that these processes are partly overlapping or that cognitive and emotional empathy, that themselves build on the ability to recognize the emotions of others, are a prerequisite for moral decisions. Furthermore, feeling similar to a person suffering might reduce emotion recognition rather than enhancing it^26^. These findings are particularly important in the context of moral dilemmas, as perspective-taking increased condemnation when unkind intentions were attributed to a protagonist, whereas (affective) empathy did not increase disciplinary responses^3^.

Brain regions associated with social cognition are the medial prefrontal cortex and bilateral temporo-parietal junction (TPJ)^27,28^. A meta-analysis on brain activation during moral decision making, empathy and theory of mind, a concept closely related to cognitive empathy^29^, found overlapping brain regions between these aspects of social cognition^30^. Particularly the dorsomedial prefrontal cortex, the bilateral TPJ and the right middle temporal gyrus were found to be activated under all three conditions, reflecting the interconnectivity of empathy, mentalizing and moral decision-making. Further brain areas related to empathy, specifically empathy for pain, were anterior insula and anterior and midcingulate cortex^31^. These regions were involved in integrating harmfulness information in judgements of the behavior in morally crucial situations^2^. The response of the right TPJ was highest for attempted harm when protagonists were condemned for their actions^32^. Regarding TRP on a neural level, Williams and colleagues found in a rather small sample that TRP depletion reduced activity in left posterior superior temporal sulcus and anterior cingulate cortex during the evaluation of the emotionality of faces^33^. These results might indicate that serotonin, decreased via TRP depletion, plays a role in modulating activity in brain areas associated with social cognition.

Thus, it is not surprising that mental disorders which are associated with altered social cognition also show altered serotonin levels^34–36^. Similar associations have been reported in ageing individuals for whom an age related decline of serotonin availability and function was hypothesized^37,38^. Consequently, TRP availability and its impact on social cognition are important to human well-being and might be a target for interventions for a broad spectrum of individuals.

The aim of this double-blinded placebo-controlled randomized controlled trial was to further elucidate effects of TRP and 5-HTP supplementation on neural and behavioral measures of social cognition in healthy participants. To allow conclusions on the long-term effects of an adapted diet, we used a 4-week L-TRP enriched diet. To find acute effects more easily, we additionally applied 5-HTP, which is almost completely metabolized into serotonin. For this, during fMRI, a task using short stories to assess moral evaluation^32^, and the reading the mind in the eyes task (RMET) to assess emotion recognition^24^ were applied. In addition, on the basis of food diaries completed over one month and urine samples, we analyzed effects of dietary TRP and basic serotonin availability in the body (inferred by urinary 5-HIAA). We expected positive effects of TRP and 5-HTP on emotion recognition and the evaluation of morally relevant behavior, as well as increased activity in areas related to social cognition. These effects should be associated to age, 5-HIAA excretion and the natural diet as we expect greater effects when people have an altered TRP availability or serotonin metabolism, e.g. in older age.

## Methods

### Participants

82 healthy participants who were randomized assigned (computerized random number list, allocated by enrollment date) to the intervention groups took part in the study (sample size was pre-determined by power analyses to detect medium effect size). The last 8 participants were allocated group-wise to match sex and age rates. Exclusion criteria were mental disorders assessed by the screening questions from the Structured Clinical Interview for DSM-IV^39^, as well as contraindications for fMRI (including hypertension). Further, food allergies or other illnesses with possible negative influence were assessed and if necessary affected individuals were excluded.

One participant was excluded due to a high 5-HIAA baseline value, one quit the study during the first session (comprehension problems), and 3 quit the study after t1 (1 longer illness, 2 without giving reasons). Demographics of the remaining 77 participants are presented in Table 1.

**Table 1:** Descriptive variables (number of cases or mean ± SD) of the 77 participants with data from at least two time points; for 5-HTP analyses all participants received 200 mg 5-HTP at t1 or t2, only order differed; only half of participants received 500 mg L-TRP enriched bars for four weeks (the other half got normal/placebo protein bars).

	All	TRP group	placebo group	*p* value TRP vs placebo
Sex: female/male	39/38	19/19	20/19	.910^a^
Age [years]	33.2 ± 10.4	33.3 ± 9.9	33.1 ± 11.1	.938^b^ (f = 0.00)
Education: number or persons with certificate of apprenticeship//number of persons with A levels or master craftsman's diploma	16//61	5//33	11//28	.104^a^
BMI [kg/m^2^]	24.1 ± 3.3	24.6 ± 3.6	23.5 ± 3.0	.148^b^ (f = 0.17)
5-HIAA baseline [mg/l]	4.7 ± 3.1	4.2 ± 2.2	5.2 ± 3.7	.140^b^ (f = 0.17)
natural TRP diet	21.7 ± 16.1	19.7 ± 14.5	23.7 ± 17.7	.323^b^ (f = 0.12)

5-HIAA: 5-hydroxyindoleacetic acid raw value was related to individual creatinine values and afterwards standardized to group mean creatinine (1045 mg/l); normal range: 2–9 mg/l.

Natural TRP diet: values refer to the sum of units (rough equivalents for consumed amount of TRP rich food items) during 28 days of natural diet (see methods and supplement for details).

^a^Chi^2^ test; ^b^ univariate ANOVA.

The study was approved by the ethics committee of the University of Heidelberg (2016-518N-MA) and conformed to the Declaration of Helsinki. It was preregistered at the German Clinical Trials Register (DRKS00010677; 25/7/2016). All participants gave written informed consent. Data acquisition was from September 2016 until March 2019 in Mannheim, Germany.

### General procedure (see also supplement)

The whole study was conducted as a double-blinded placebo-controlled trial. The primary outcomes were behavioral and neural measures of social cognition. Participants had three appointments, each comprising laboratory testing and an MRI session. All measures of each participant were planned on the same weekday and at the same time to reduce possible influences of circadian rhythms, working hours and recreational activities.

At t1 and t2, all participants got either 5-HTP or placebo in a randomized cross-over design (double-blinded), age and sex were counterbalanced between groups (see also supplemental Table 1A,B). For this, they took cellulose capsules containing either 200 mg 5-HTP (extracted from *Griffonia simplicifolia*) or placebo (cellulose and mannitol) with a 200 ml mixture of fruit juice (15–20%) and water. The capsule was given 1.5 h before placing the participant inside the scanner (mean time capsule taken until first task started: 110 ± 11 min for both conditions). To avoid possible influences of 5-HTP on baseline 5-HIAA, participants gave a urine sample before taking the capsule. 200 mg 5-HTP were chosen as similar doses were reported previously to have an effect in depressed individuals (often higher doses are used in these studies as well; as we included healthy participants we decided on a dose at the lower end of the spectrum of doses used before, for an overview of different doses used see:^40^).

**Figure 1:**
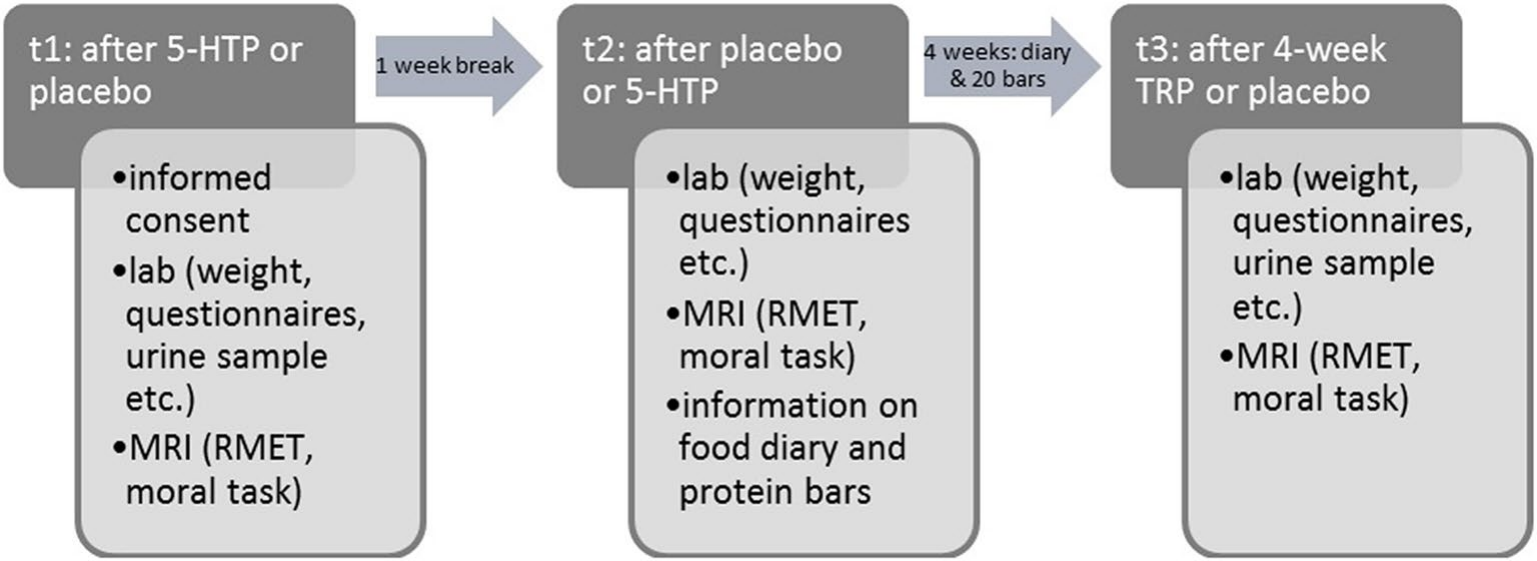
Schematic overview of the study; t1/t2: first part with acute 5-HTP challenge in cross-over design, all participants completed both conditions but at different time points; t3 and placebo condition from t1 or t2: second part with 4-week TRP enriched diet or placebo, every 40 participants completed one of the conditions (mixed between subject design); RMET: reading the mind in the eyes task.

After completion of the first part of the study (t1 to t2) the second part of the study using a mixed between-subject design started using the placebo condition from the first part as a baseline. Participants filled in food diaries and ate protein bars over one month. Half of them received placebo/normal protein bars (0.39 mg L-TRP) and the other half got the same protein bars injected with additional 500 mg L-TRP (randomized, double-blinded; age and sex counterbalanced, see also supplemental Table 1). 500 mg TRP were chosen as this is close to the recommended daily dose for many normal weight adults and we aimed at simulating an adapted diet which individuals could reach via changing eating habits. Participants were instructed to eat one bar weekdays in the morning. At t3, all measurements were taken for a last time (study scheme Fig. 1).

Urine samples were analyzed for 5-HIAA (liquid chromatography-mass spectrometry) and creatinine by a commercial laboratory (MVZ Labor Dr. Limbach, Heidelberg, Germany). Creatinine was measured with a double determination to relate 5-HIAA values to urine concentration as this depends highly on e.g. fluid intake and activities. Therefore, 5-HIAA raw values were related to individual creatinine values and afterwards standardized to group mean creatinine (1045 mg/l). For collecting further anthropometric data a body composition scale (Omron body composition monitor BF511) was used.

### Tasks and questionnaire (see also supplement)

**Figure 2:**
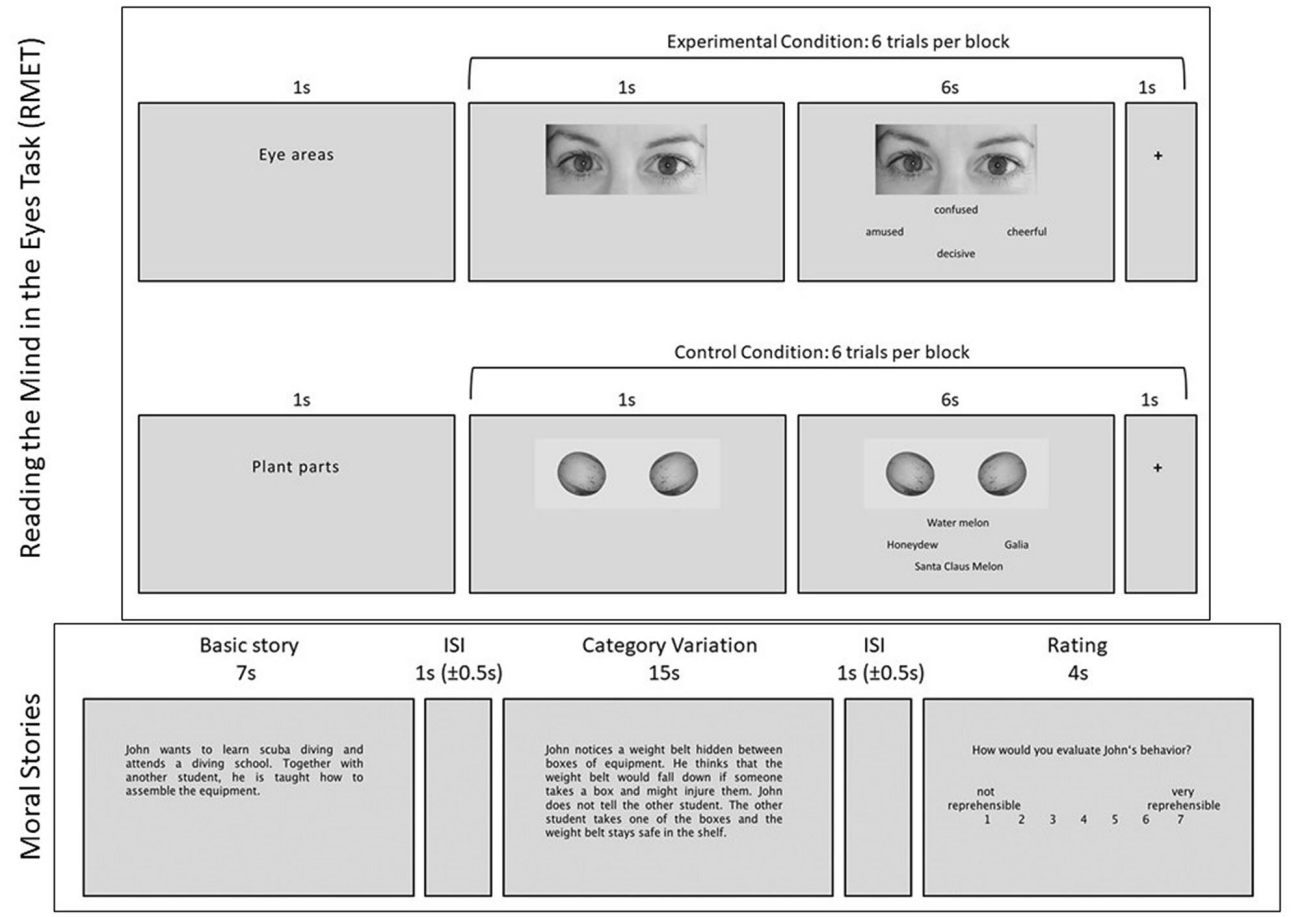
Example trials of both tasks; upper: reading the mind in the eyes task (RMET); lower: moral judgement task showing the NP version of the used training item (every participant had one training item per task which was not included in analyses, these two items were the same for all participants).

The RMET^24^ is designed to assess mentalizing and emotion recognition. It involves inferring the mental state of a person from photographs of 36 eye regions. As a contrast baseline we used 36 greyscale pictures of plants (as the photographs in the original RMET are in greyscale). Stimuli were presented on a grey background in the center of the screen with four mental state terms or four plant names below a picture (see Fig. 2). In every trial, one target label and three distractors were simultaneously shown. The participant’s task was to select the correct mental state or the correct name of the plant as fast as possible. Performance was determined by number of correct answers. The RMET items 1, 3, 6, 10, 12, 13, 15, 16, 18, 20, 21, 23, 25, 27, 28, 29, 30, 31 were rated as being more positive and 2, 4, 5, 7, 8, 9, 11, 14, 17, 19, 22, 24, 26, 32, 33, 34, 35, 36 as more negative (for more details please see supplement). The same 72 pictures were presented in three different orders (one for every session).

In the moral judgement task^32^ participants read stories describing an action and an outcome, together with the extent of relevant information available to the protagonist (see Fig. 2). Participants are asked to rate the moral reprehensibility of the behavior of the protagonist whereby protagonists either do or do not foresee a possible negative outcome of their behavior. In other words, e.g. a protagonist who believed in producing a negative outcome and actually produced a negative outcome did so knowingly. This 2 × 2 design results in four different scenario categories: negative belief/negative outcome (NN, “successful attempt to harm”), negative belief/neutral or positive outcome (NP, “attempted harm”; as introduced this was the category of main interest), neutral or positive belief/negative outcome (PN, “accidental harm”) and neutral or positive belief/neutral or positive outcome (PP, “no harm”). Participants judged the moral reprehensibility of the protagonist’s action on a 7-point Likert-scale ranging from 1 (not reprehensible at all) to 7 (absolutely reprehensible). In total, participants read and judged 24 stories at every time point at the end of the MRI session, which means overall the same 72 stories in the whole study for every participant. For this, the number of stories in the original moral task was split into three comparable parts. Consequently, participants read new stories at every time point at which every basic story was presented two times including two different scenario categories (matched between the three time points). Only two instead of all four categories for each basic story were selected, because presenting all four might have been too repetitive for participants possibly affecting their attention to the differences.

The Empathy Quotient (EQ; Baron-Cohen & Wheelwright, 2004) was used to assess self-rated empathy including three subscales^41^: cognitive empathy, emotional reactivity, and social skills. The EQ was also assessed thrice at which the placebo condition at t1 or t2 served as a baseline for analyses of the effects in the one-month TRP supplementation intervention period and natural TRP analyses. 5-HTP effects on EQ were not analyzed as one week was seen as a too short time period to detect changes.

### fMRI data acquisition and analyses

The fMRI experiment was conducted using scanner built-in VisuaStim video goggles (Resonance Technology Inc., Northridge, USA) and the Presentation software (version 18.1; www.neurobs.com) for stimulus presentation. Participants responded with a response device (Current Designs, Inc., Philadelphia, USA). Stimuli were presented for 7 s during the RMET (1 s picture only, 6 s picture with answer selection) and for 22 s during the moral task (7 s basic story, 15 s category variation) with additional 4 s for response. Trials in the moral task were separated with a variable inter stimulus interval (ISI) of 1 s on average (jitter 0.5 s–1.5 s). During the RMET an ISI of 1 s was used with the presentation of a fixation cross in the position where the next picture would show (see Fig. 2).

304 (RMET) and 359 (moral task) T2* weighted EPI images (TR = 2 s, α = 80°, TE = 28 ms) with 33 slices (slice thickness 3 mm, voxel 3 × 3 × 3 mm^3^, FOV 192 mm^2^) were recorded with two identical 3T Trio Tim scanners with 12 channel head coils (Siemens Healthineers, Erlangen, Germany). Further, we collected high-resolution three-dimensional T1 weighted anatomical images (MPRAGE; TR = 2.3 s, α = 9°, TE = 3.03 ms) with 192 slices (slice thickness 1 mm, voxel 1 × 1 × 1mm^3^, FOV 256mm^2^). Heart rate and respiration rate were sampled at 50 Hz with scanner built-in equipment (PMU Wireless Physio Control, Siemens Healthineers, Erlangen, Germany). Half of the sample was scanned on one scanner and the other half on the second (all measurements per participant on the same).

The first 4 images of each task were discarded. Data were corrected for physiological artefacts including a high-pass filter of 1/512 Hz using the Aztec software tool^42^, and for small head movements by wavelet despiking using the BrainWavelet toolbox^43^. FMRI analyses were conducted with SPM12 v7219 (Wellcome Trust Centre for Neuroimaging, University College London, UK) running on MATLAB R2017a (The MathWorks Inc., Natick, USA). Preprocessing included segmentation of the MPRAGE and registration to the SPM12 TPM templates, coregistration of the functional images to the individual MPRAGE, motion correction, slice time correction, normalization of the functional images with parameters derived during MPRAGE segmentation, and smoothing with a 9 mm Gaussian kernel. For first level analyses, a GLM was set up with the onsets of the stimuli (RMET: picture presentation contrast eye regions > plants; moral task: both text presentations) as regressors of interest and the 6 movement parameters (all participants: translation < 3 mm, rotation < 3°) derived from realignment and the response clicks as regressors of no interest. Afterwards, maps were corrected for the baseline measurements which were the corresponding placebo conditions (X − baseline) to get change values for each individual. For the second level analyses on group level (5-HTP vs 0 or L-TRP vs placebo) we used these change values and included age, sex, baseline 5-HIAA, and the order of 5-HTP and placebo administration as covariates. To account for possible effects of the two scanners, we did an additional analysis with the scanner number as covariate. Further, we conducted a parametric modulation for the NP stories with the behavioral data of those.

### Food diary analyses

A list of foods rich in TRP was used to extract a natural TRP score from the food diaries. The Food Composition Database SR-26 of the United States Department of Agriculture (https://ndb.nal.usda.gov/ndb/search/list via GitHub 2/2016) was used as a basis and pre-selected for foods for which all relevant amino acids and macronutrients were reported and which contain at least 200 mg of TRP per 100 g. Both TRP/LNAA and TRP/tyrosine ratios were computed and used as sorting criteria. For both sorting criteria, the 25 highest were selected and due to their great overlap, the final list consists of 29 TRP-rich foods (see supplement).

Most participants did not provide information concerning the exact amount of foods, which is why food quantities were estimated via serving sizes as defined by the German Federal Center for Nutrition with one serving referring to one unit (small serving: 0.7 units, large: 1.3, trivia: 0.1). The natural TRP score is calculated as sum of units over all 28 days. In addition, all food items were grouped into categories, e.g. meat or legumes, and summed up.

### Combinatory analyses

We conducted rmANCOVAs with age, sex, 5-HIAA baseline values, BMI, and order (5-HTP at t1 or t2) as covariates, regressions and correlations with IBM SPSS 25 (SPSS Inc., Chicago, Illinois, USA). Effect sizes were calculated with G*Power 3.1.9.2^44^. We used age as a covariate as we were especially interested in the effect of age in this research project. The other four covariates were mainly used to control for possible influencing effects (sex: although we matched sex between groups this could have additional influence; BMI: control for differences in body metrics as we had the same doses for every participant; 5-HIAA baseline values: account for possible influence of present serotonin status of participants; order: account for possible order effects).

rmANCOVAs detecting a possible 5-HTP effect were conducted with values from t1 and t2 (RMET: one rmANCOVA; moral task: one rmANCOVA for each category). rmANCOVAs detecting a possible TRP effect were conducted with t3 values and the placebo condition values from t1 or t2 as a baseline (RMET: one rmANCOVA; moral task: one rmANCOVA for each category). Please see also supplemental Table 2 for all included variables in every rmANCOVA.

Further, we extracted the first eigenvariates (corrected for covariates) of the brain regions significant in MRI results as marker of change in brain activity. Food diary data was used in correlational and 3 regression analyses (RMET positive emotions, RMET negative emotions, NP story rating) where baseline values (the values from the placebo condition at t1 or t2) were included as additional predictors.

## Results

### Differences in behavior: main results

#### Acute effects of 5-HTP supplementation (t1 and t2)

Emotion recognition (RMET) was not related to 5-HTP intake (F_1,70_ = 0.18, *p* = 0.670, f = 0.05), but there was a significant effect of the covariate age (F_1,70_ = 22.12_,_ *p* < 0.001, f = 0.56; negative association of age with performance; see supplemental Table 2 for results including all covariates and supplemental Table 3 for time effects).

**Figure 3:**
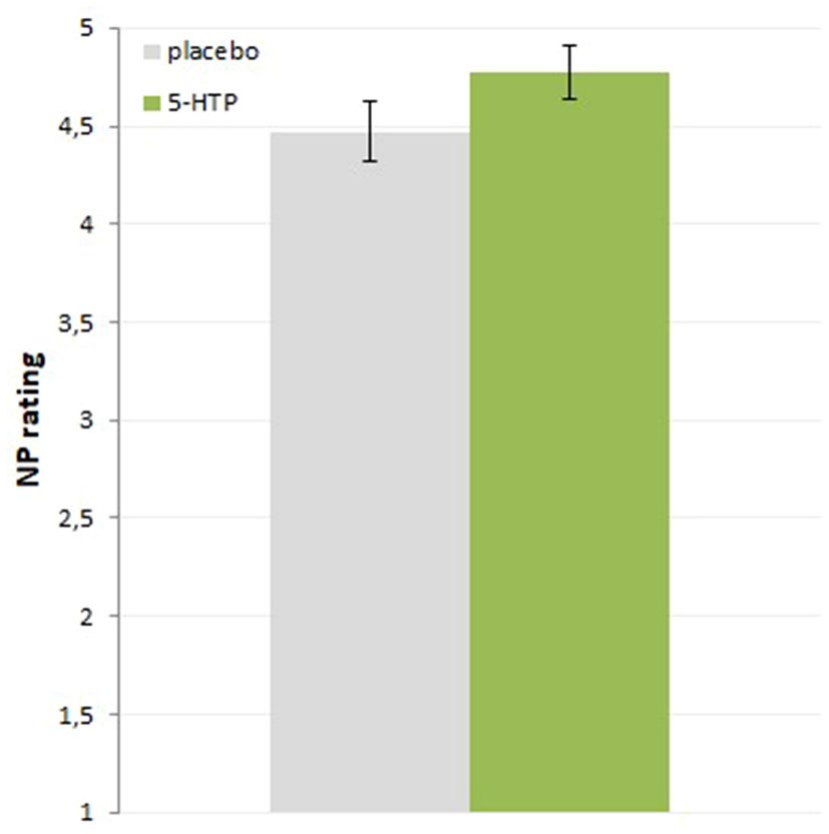
Moral judgement task behavioral effect of substance on the reprehensibility ratings (1–7, 7 very reprehensible) of the moral stories with a good ending although the protagonist behaved badly (NP condition), significantly higher NP rating after 200 mg 5-HTP intake compared to placebo (n = 77; cross-over design); means with SE; see baseline values of placebo condition for all 4 categories in supplemental Fig. 1.

With respect to the moral judgement task, even after controlling for covariates there was a main effect of substance showing that the behavior of the protagonist in NP stories was rated as more reprehensible with 5-HTP (F_1,70_ = 7.45, *p* = 0.008, f = 0.33, Fig. 3; baseline 5-HIAA effect: F_1,70_ = 4.91, *p* = 0.030, f = 0.27, positive association between 5-HIAA values and NP ratings). The ratings of the other story categories showed no significant substance effect (PP: F_1,70_ < 0.01, *p* = 0.976, f = 0.00; PN: F_1,70_ = 0.48, *p* = 0.490, f = 0.08; NN: F_1,70_ = 0.66, *p* = 0.421, f = 0.10).

#### 4-week TRP enriched diet and natural/dietary TRP (t3 and placebo/baseline condition from t1 or t2)

Regarding the RMET, no significant effects of 4-week TRP supplementation on emotion recognition were found (F_1,65_ = 0.08_,_ *p* = 0.781, f = 0.03). Importantly, with respect to the TRP-enriched diet there was a significant effect of the covariate age on emotion recognition (F_1,65_ = 22.28_,_ *p* < 0.001, f = 0.59; BMI effect: F_1,65_ = 4.36_,_ *p* = 0.041, f = 0.26, higher BMI lower TRP ‘effect’; other covariates see supplemental Table 2). Worse emotion recognition was found with higher age (pos.: r = − 0.30, *p* = 0.004; neg.: r = − 0.62, *p* < 0.001) which disappeared for positive emotions with TRP (r = 0.04, *p* = 0.407). In addition, a small but non-significant effect of natural TRP regarding the recognition of positive emotions was identified (full model T_7,56_ = 6.63_,_ *p* < 0.001, R^2^ = 0.39; natural TRP T_7,56_ = 1.89_,_ *p* = 0.064; baseline emotion recognition effect: T_7,56_ = 5.52_,_ *p* < 0.001).

Regarding the moral task, individuals who had received TRP rated the protagonist’s behavior in NP stories not significantly more reprehensible than those who received placebo (F_1,64_ = 0.53, *p* = 0.470, f = 0.09; 5-HIAA baseline effect: F_1,64_ = 9.15, *p* = 0.004, f = 0.38, higher 5-HIAA values related to lower NP ratings. Other story categories showed also no significant effects of substance (PP: F_1,64_ = 2.12, *p* = 0.152, f = 0.18; PN: F_1,64_ = 2.25, *p* = 0.138, f = 0.19; NN: F_1,64_ = 0.61, *p* = 0.438, f = 0.10). As for the moral task behavioral effects were only found for NP stories (which was also the category of main interest), all following analyses were only applied to these. A significant effect of natural TRP on the rating of NP stories could not be found (T_7,62_ = 0.35, *p* = 0.727), whereupon baseline NP ratings (NP ratings before the food diary was filled in which means during the placebo condition either at t1 or t2; T_7,62_ = 6.78, *p* < 0.001), 5-HIAA (T_7,62_ = − 3.08, *p* = 0.003) and order (T_7,62_ = − 3.49, *p* = 0.001) were significant predictors.

### Differences in brain activation

For the RMET, the main effect (eyes > plants) found at *p*_FWEcor._ < 0.05 showed activation in e.g. the middle and inferior frontal gyrus and superior temporal sulcus. The main effect for moral task NP stories at *p*_FWEcor._ < 0.05 showed positively activated brain regions comparable to those found before (middle/medial prefrontal cortex, precuneus, parahippocampal gyrus) but the effects especially in the TPJ were much smaller (peak voxel *t* = 2.34).

#### Brain activation effects regarding TRP and 5-HTP supplementation

**Figure 4:**
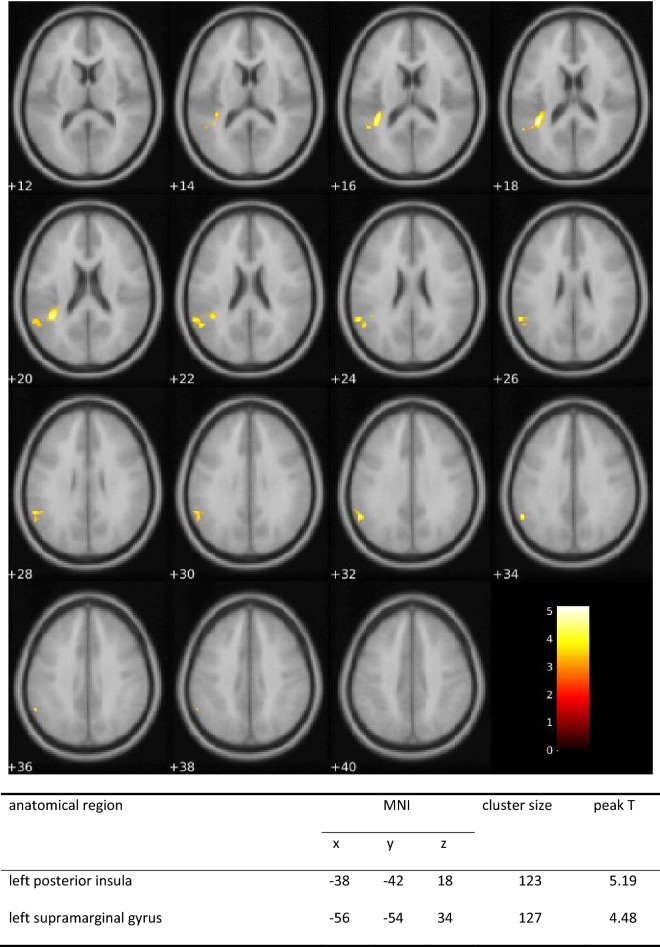
Moral task fMRI effects of substance: Positive activation change of posterior insula and supramarginal gyrus of the left hemisphere after one month of TRP-enriched diet (compared to placebo) during reading morally bad behavior without negative consequences for the potential victim (NP condition; axial view; colors indicate *t*-values; cluster defining threshold *p*_unc._ < .001, minimum cluster size 100 voxel, cluster *p* < .05 FWE_cor._)

5-HTP showed no neural effects during RMET or moral task at a cluster defining threshold *p*_unc._ < 0.001 with a minimum cluster size of 100 voxel. 4-week TRP supplementation was also not related to any neural activation differences compared to placebo in the RMET (*p*_unc._ < 0.001, k ≥ 100). For the moral task, a substance effect for NP stories was found. Reading these stories was associated with more activity in posterior insula and supramarginal gyrus in the TRP group. This pattern of results did not change when including the scanner as covariate (Fig. 4).

#### Additional findings: combination of fMRI and behavioral data and different behavioral data

The parametric modulation of fMRI time series during reading NP stories and the NP ratings did not show any significantly active brain areas for TRP compared to placebo. The correlation of the activity changes in the two significant clusters and the changes in reprehensibility ratings did not show significant relationships (supramarginal gyrus: r = 0.03, *p* = 0.414; insula: r = − 0.05, *p* = 0.351).

After 5-HTP, better recognition of positive emotions and worse recognition of negative emotions were associated with higher reprehensibility ratings (pos.: r = 0.27, *p* = 0.008, neg.: r = − 0.22, *p* = 0.027; corrected for baseline values; supplemental Fig. 2). After TRP, better recognition of positive emotions might be associated with higher reprehensibility ratings (pos.: r = 0.23, *p* = 0.094, neg.: r = 0.06, *p* = 0.373; corrected for baseline values).

### Exploratory analyses on factors possibly influencing TRP effects

An additional analysis of the effect of TRP on emotion recognition in higher age (40 +) group showed a significant positive effect (F_1,13_ = 5.05_,_ *p* = 0.043, f = 0.62). Further, a higher BMI was associated with less dietary/natural TRP (r = − 0.23, *p* = 0.035) and lower excretion of 5-HIAA (r = − 0.22, *p* = 0.031). Meat consumption (different types were not considered separately) correlated negatively with urinary 5-HIAA (r = − 0.42, *p* < 0.001), whereas legumes showed a positive, but insignificant correlation (r = 0.19, *p* = 0.059).

Regarding the empathy quotient (EQ), TRP had no effects on the total scale (F_1,66_ = 0.20, *p* = 0.665, f = 0.05) or sub scores (cognitive empathy: F_1,66_ = 0.10, *p* = 0.748, f = 0.04; emotional reactivity: F_1,66_ = 0.11, *p* = 0.746, f = 0.04; social skills: F_1,66_ = 1.00, *p* = 0.321, f = 0.12). There seemed to be no effect of time for the total EQ scale (f = 0.19; *p* = 0.111). Cronbach’s α of the EQ at t3 was 0.72 for the total scale, 0.93 for cognitive empathy, 0.86 for emotional reactivity, and 0.63 for social skills.

## Discussion

The aim was to identify effects of tryptophan on social cognition, in particular on emotion recognition and moral evaluation varying with a 4-week TRP enriched diet, acute 5-HTP intake, and dietary TRP. These effects could be helpful in creating tailored diets for some vulnerable individuals, e.g. in aging.

The most prominent differences were found for the moral stories in which the protagonist behaved badly although knowing that it might have a negative outcome but this failed to materialize. Normally, people are more outcome oriented^45^ but when participants took 5-HTP, they rated the behavior as more reprehensible. This could be seen as a stronger sanction of negative behavior despite a non-harmful outcome. In line with this interpretation, increased activation in posterior insula and supramarginal gyrus could be found after the 4-week TRP-enriched diet. The supramarginal gyrus, as a part of the TPJ, is associated with perspective-taking and empathy, and integration of related information. This could indicate that TRP and 5-HTP might increase empathy with the potential victim. Whereas self-rated empathy did not change with TRP, some associations of emotion recognition (as an indicator of cognitive empathy) with the reprehensibility ratings after receiving 5-HTP give us reason to speculate that the effect of TRP might depend on the specific cognition related empathy-processes at hand. This would fit to the findings of other authors, who propose perspective-taking in moral situations as an important factor in their evaluation^3^ or report that the feeling of guilt was specially elevated in highly empathic participants under TRP depletion when completing a task on social situations including unjust harm^46^.

However, in contrast, the subscales of the empathy questionnaire did not show significant changes with TRP either. This might be a problem of the self-rating procedure or suggest that the effects are not primarily related to empathy or perspective-taking. Other authors found the TPJ to be active during moral judgement when encoding beliefs and integrating information^32,47^. Brain regions related to empathy for pain were also previously found to be involved in integrating harmfulness information^2^. For that reason, TRP or 5-HTP intake might improve serotonin availability in the brain and the integration of all available information and therefore accentuate the relevance of the behavior and belief of the protagonist in contrast to the outcome. Curiously, both, the parametric modulation of the ratings with fMRI time series and the correlation between brain activation and the ratings revealed no significant associations. This could be related to a power (TRP smaller n) or dosage problem but might also indicate that the better integration of information on e.g. beliefs does not automatically lead to the use of this information during evaluation. In short, we speculate that our fMRI results point more to an altered information integration by TRP but further studies are needed to get deeper insights here. Importantly, brain activation was corrected for covariates like 5-HIAA which might has reduced possible associations as 5-HIAA levels explained a significant part in the found behavioral effects.

As urinary 5-HIAA is a serotonin metabolite, its excretion indicates that there was a sufficient amount of TRP or 5-HTP in the body to metabolize serotonin. Several processes can influence 5-HIAA levels e.g. the reuptake of serotonin, melatonin production or external variables like alcohol or caffeine^48,49^. Additionally, the TRP requirement in the body can vary with some diseases or BMI^50,51^ so that the availability in the brain is reduced when more TRP is metabolized in other parts of the body e.g. due to baseline deficiencies. Also in our sample, a higher BMI was associated with lower excretion of 5-HIAA. Therefore, even in healthy populations weight might have effects on TRP availability. All participants got the same dosage, which was necessitated by the double-blind protocol, and considered appropriate, given that all participants were healthy and of normal weight. Nevertheless, it might have been suboptimal for some of the participants and on average especially for TRP the dosage might be too low. Furthermore, participant’s natural diet showed that people with higher BMI consumed less TRP. This could be an additional reason why 5-HIAA levels are lower or this even intensifies a possible lack of TRP in these individuals. In addition, as the placebo bars contained also protein but only a very small amount of TRP, the effects might partly be additionally triggered by other LNAAs. The influence of meat consumption (often tyrosine rich) versus legume consumption (often TRP rich) on 5-HIAA levels may hint to this point, too. But of course several other dietary components like carbohydrates play important roles in this metabolism as well and should be taken more into account. The same is valid for including e.g. urinary kynurenine values to better account for other serotonin metabolites. As some authors found a shift in the tryptophan metabolism from serotonin to kynurenine for different mental disorders^52^, this might be also of importance in a healthy population especially for vulnerable individuals. As these values were not available in this study, this can be seen as a limitation, which should be addressed in the future.

We find first hints for a positive influence of TRP on recognition of positive emotions and of the better recognition on reprehensibility ratings which was even more pronounced for 5-HTP. The opposite effect, worse emotion recognition after TRP depletion, was found before^53^ but for the present whole sample, the positive effect of TRP on emotion recognition was not significant. In a larger sample habitual TRP consumption was related to emotion recognition^54^. Importantly, in line with our hypotheses and research proposing an altered serotonin metabolism in higher age^37^, we found age to be a significant contributing factor to TRP and 5-HTP effects. In older participants, especially the recognition of positive emotions was improved after TRP. This might point to a compensatory effect of TRP in older individuals who could improve social cognitive processing with a TRP-rich diet. Of course, those results should be seen critically and need replication, especially also in samples with older participants. Accordingly, also genetic and epigenetic features seem to play an important role in this pathway^55^, the monoamine oxidase A high-activity allele together with the degree of methylation at a promoter CpG site on the tryptophan hydroxylase 2 gene explain significant quantities of variance in the RMET performance, pointing to people at risk might profit more from TRP supplementation or a TRP rich diet. This and the possible age-related effect could imply that especially people with a lack of TRP or other metabolic variations which have influence on the serotonin metabolism are those who profit most which is why effects are harder to detect in mixed samples including also individuals with a normal serotonin metabolism.

Importantly, analyses of natural TRP on moral stories showed an effect of the time point of 5-HTP administration which could imply related altered learning and memory effects. Such effects could be found before in animals^56^ and multiple sclerosis patients^57^. As ratings might be influenced by the wording and form of questions asked when evaluating moral behavior^45^ this might have had a further influence on learning processes (e.g. asking for reprehensibility vs. permissibility). However, we aimed at reducing this influence as much as possible by structuring the stories following uniform rules and using the same question with a rating scale for all stories.

An important limitation of the study is that some effects were only significant in one condition and not in both (TRP and 5-HTP). Due to the different designs of the two parts of the study, we had 77 participants for the 5-HTP part but only 36 for the TRP part. This might have led to power problems especially in the latter one. Additionally, for detecting TRP effects the tasks were applied thrice and for the 5-HTP effect only twice which might had an influence too. This could explain the non-finding of behavioral changes with TRP but cannot explain that possible 5-HTP effects were not detected on the chosen threshold with fMRI. It should be a point of future studies if this is also true for larger sample sizes, higher 5-HTP doses or another application of the tasks.

In sum, even in healthy individuals who have lower TRP availability or an altered serotonin metabolism (e.g. older age, higher BMI, nutrition), TRP or 5-HTP supplementation seems to have a positive influence on the recognition of positive emotions and the integration of information in moral judgements. Besides above mentioned factors, the 5-HIAA level, indicative of serotonin status, might be an additional rough measure for individualized diet recommendations. Notably, future studies should use not only individualized dosages of TRP, but also include more biological and psychological factors to deepen our knowledge of this important pathway influencing our social behavior, and physical and mental health.

## Supplementary Information

Supplementary Information.

## Data Availability

The datasets generated and analyzed during the current study are available from the corresponding author on reasonable request.
