# Supplementary material for: Tryptophan-enriched diet or 5-hydroxytryptophan supplementation given in a randomized controlled trial impacts social cognition on a neural and behavioral level
Source: Sci Rep. 2021 Nov 4;11:21637. doi: 10.1038/s41598-021-01164-y (PMC8568973; doi:10.1038/s41598-021-01164-y)
Supplement: Supplementary file 1 — Supplementary Information. [file 41598_2021_1164_MOESM1_ESM.pdf]

Supplemental material

Tryptophan-enriched diet or 5-hydroxytryptophan supplementation given in a randomized controlled trial impacts social cognition on a neural and behavioral level

Zamoscik V, Schmidt SNL, Bravo R, Ugartemendia L, Plieger T, Rodríguez AB, Reuter M, Kirsch P

General procedure details

The procedure at the time points (t1+t2) was completely the same except the explanation of the study, the approval to the informed consent and urine sample collection was only before t1, and instructions for the food diary and the 20 protein bars were given only after t2.
The timing for 5-HTP administration was chosen because 1) the first peak of 5-HTP in the body occurs at about 1.5h after 5-HTP consumption [1,2], and 2) according to the pharmacist who prepared the capsules, the capsule shell dissolves about 10 minutes after ingestion.
The bars schedule was used as we expected participants to follow the instructions with greater ease during regular weekdays, and to prevent an oversupply of TRP, which could influence tyrosine crossing the blood brain barrier, resulting in changed dopamine levels as an unwanted effect.

Because of the respective expiry dates, we used one charge of 5-HTP capsules, but three charges of protein bars over the course of the whole study. The charge number was used as a covariate in exploratory analyses to account for possible effects of different production / delivery dates but we did not find any effects of charge number.

Supplemental table 1A: Matching table; age and sex were counterbalanced between double-blinded groups (A and 1 refer to the placebos; B: 200mg 5-HTP, 2: 500mg L-TRP; example: AB1 means placebo at t1, 5-HTP at t2 and placebo bars before t3)

GROUP	WOMEN (ALL INCLUDING SOME WITH INCOMPLETE DATA /ALL WITH ALL DATA)	MEN (ALL INCLUDING SOME WITH INCOMPLETE DATA /ALL WITH ALL DATA)	MEAN AGE [YEARS]
AB1	10	12/10	33.32
AB2	10/9	10/9	33.33
BA1	10	10/8	33.83
BA2	10	10	33.25

Supplemental table 1B: Descriptive variables (number of cases or mean ± SD) of all participants who received 200mg 5-HTP at t1 or t2, separated by order

	T1 PLACEBO	T1 5-HTP	P (F)
SEX: F/M	19/19	20/19	.910
AGE [YEARS]	33.11 ± 10.83	33.28 ± 10.18	.941 (.00)
EDUCATION	6//32	10//29	.287
BMI [KG/M^2]	23.76 ± 3.58	24.35 ± 3.11	.442 (.09)
5-HIAA BASELINE [MG/L]	5.23 ± 3.88	4.25 ± 1.97	.167 (.16)
NATURAL TRP	19.39 ± 14.66	24.02 ± 17.41	.248 (.15)

1 Magnussen, I. & Nielsen-Kudsk, F. Pharmacokinetics of intravenously administered L-5-hydroxytryptophan in man. *Acta Pharmacol Toxicol (Copenh)* **44**, 308-314, doi:10.1111/j.1600-0773.1979.tb02335.x (1979).

2 Westenberg, H. G., Gerritsen, T. W., Meijer, B. A. & van Praag, H. M. Kinetics of l-5-hydroxytryptophan in healthy subjects. *Psychiatry Res* **7**, 373-385, doi:10.1016/0165-1781(82)90074-9 (1982).

Tasks details
Before data acquisition, all RMET items were independently rated by 3 psychologists (2 female, 1 male) in regard to a more positive or more negative valence as we needed different orders of the task for a repeated presentation and did not want to show too many items of „similar“ valence in a row. Interrater reliability was above .9, few items had to be discussed at which we had chosen to use the rating of the majority as this in addition lead to every half items of positive and negative valence (18 each). In detail, the items 1, 3, 6, 10, 12, 13, 15, 16, 18, 20, 21, 23, 25, 27, 28, 29, 30, 31 were rated as being more positive and 2, 4, 5, 7, 8, 9, 11, 14, 17, 19, 22, 24, 26, 32, 33, 34, 35, 36 as more negative. Before data analyses, we decided to use the valence classification also in some analyses and asked two more persons (1f, 1m; research assistants) about their ratings, those were highly overlapping with the initially ones.

List of TRP rich food items:

whey products, mozzarella, cheddar, cocoa, cashew, chia seeds, linseed, sesame, pumpkin seeds, sunflower seeds, pistachios, almonds, hemp seeds, wheat bran, oat bran, Kellogg's All Bran, kidney beans, white beans, soy, eggs, salmon, halibut, crayfish, shrimps, crabs, lobster, turkey, pancreas, liver

Example calculations of natural TRP via food diaries:

Participants were instructed to indicate if they ate more or less than one normal serving size (no number or 1 indicated one normal serving size).

Original text by participant for his/her breakfast: “8:30 1 Kaffee, 1 Toast Cheddar”

Original text by participant for his/her dinner: “Spaghetti mit Pesto Rosso, Limo, Espresso, Zartbitterschoko”

This was converted into a format readable for our matlab script and translated into English:

coffee, toast, cheddar

pasta, pesto, lemonade, espresso, chocolate dark

Chocolate dark and cheddar counted as TRP rich food items. If this person ate only these 2 portions TRP-rich foods that day, she/he would receive a score of 2 for that day. Importantly, to get the natural TRP score used for the reported analyses, all such items were summed up over all 28 days.

Additional results and additional information/display of results:

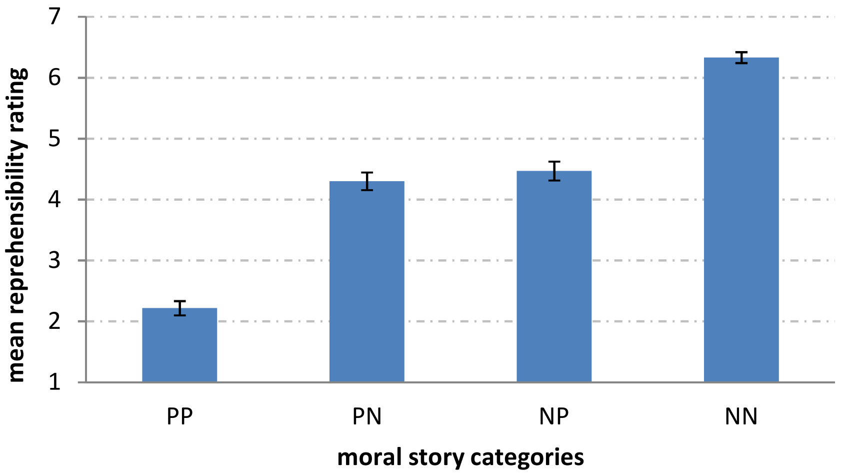

Supplemental figure 1: Mean reprehensibility ratings of baseline placebo condition for all 4 moral story categories

Supplemental table 2: rmANCOVAs for RMET emotion recognition and moral judgement task reprehensibility ratings with all included variables; order means order of 5-HTP and placebo condition in study part one as the placebo condition served as baseline for TRP analyses

		**F**	***P***
**5-HTP**			
emotion recognition	5-HTP	0.18	.670
	age	22.12	.000
	sex	1.34	.252
	5-HIAA baseline	0.78	.379
	BMI	1.44	.234
NP rating	5-HTP	7.45	.008
	age	1.59	.212
	sex	0.96	.332
	5-HIAA baseline	4.91	.030
	BMI	0.21	.645
PP rating	5-HTP	< 0.01	.976
	age	1.51	.223
	sex	0.23	.633
	5-HIAA baseline	0.15	.697
	BMI	0.07	.798
PN rating	5-HTP	0.48	.490
	age	0.68	.413
	sex	0.59	.447
	5-HIAA baseline	1.45	.233
	BMI	0.15	.697
NN rating	5-HTP	0.66	.421
	age	0.40	.531
	sex	2.53	.116
	5-HIAA baseline	1.31	.256
	BMI	1.80	.184
**TRP**			
emotion recognition	TRP	0.08	.781

	age	22.28	.000
	sex	3.16	.080
	5-HIAA baseline	0.46	.499
	BMI	4.36	.041
	order	0.62	.434
NP rating	TRP	0.53	.470
	age	1.68	.199
	sex	2.88	.094
	5-HIAA baseline	9.15	.004
	BMI	0.56	.455
	order	1.12	.294
PP rating	TRP	2.12	.152
	age	1.78	.187
	sex	0.01	.945
	5-HIAA baseline	0.25	.616
	BMI	1.56	.217
	order	0.01	.943
PN rating	TRP	2.25	.138
	age	0.13	.716
	sex	0.10	.751
	5-HIAA baseline	1.02	.316
	BMI	0.44	.512
	order	2.17	.146
NN rating	TRP	0.61	.438
	age	0.38	.538
	sex	7.86	.007
	5-HIAA baseline	0.01	.918
	BMI	0.01	.905
	order	8.76	.004

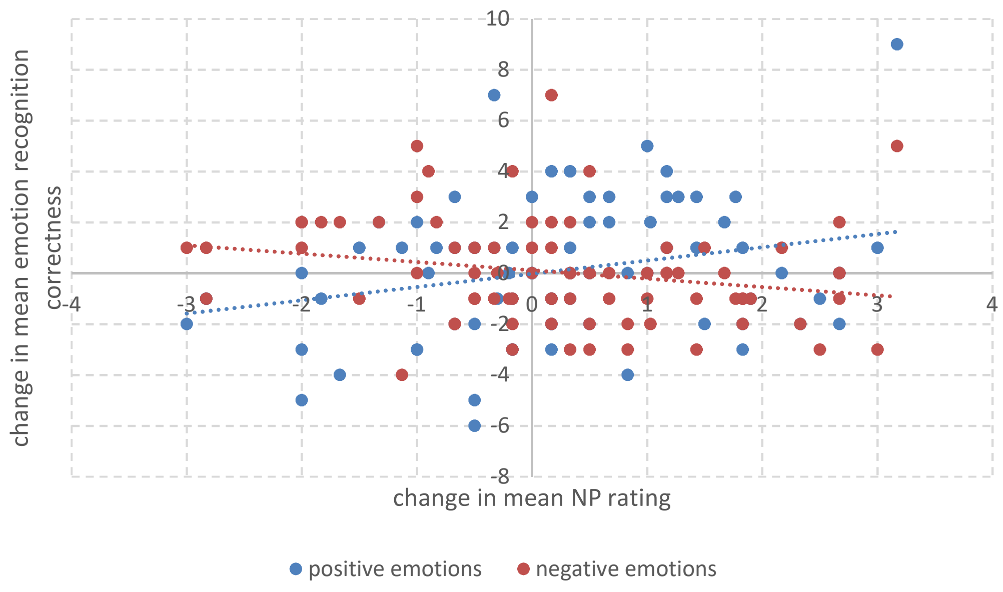

Supplemental figure 2: 5-HTP effect: significant relations of mean change values of emotion recognition and NP reprehensibility ratings (change values calculated: 5-HTP condition – placebo condition)

Supplemental table 3: time effects of moral task PP, PN, NP, NN ratings and RMET emotion recognition performance (means with SD)

	EMOTION RECOGNITION	PP RATING	PN RATING	NP RATING	NN RATING
**T1**	22.66 ± 4.30	2.22 ± 1.13	4.51 ± 1.36	4.17 ± 1.06	6.19 ± 0.67
**T2**	23.14 ± 4.20	2.20 ± 0.87	3.99 ± 1.28	5.04 ± 1.33	6.51 ± 0.75
**T3**	23.21 ± 4.44	2.10 ± 1.03	3.52 ± 1.61	5.03 ± 1.31	6.23 ± 0.77
